# PROTOCOL: Interventions designed to improve financial capability by improving financial behavior and financial access: A systematic review

**DOI:** 10.1002/cl2.1020

**Published:** 2019-07-19

**Authors:** Julie Birkenmaier, Brandy Maynard, Youngmi Kim

**Affiliations:** ^1^ Saint Louis University College of Public Health and Social Justice School of Social Work St. Louis Missouri; ^2^ Virginia Commonwealth University School of Social Work Richmond Virginia

## 1 BACKGROUND

### 1.1 The problem, condition or issue

Financial capability, defined as “*a consumer's ability to apply financial knowledge and perform desirable financial behaviors to achieve financial well‐being*” (Xiao & O'Neill, 2014, November), is gaining attention in practice and public policy. Today's financial context is more complicated in that it offers more types of financial products and services from which to choose than in previous generations, and people have greater responsibility for making some financial decisions with long‐term ramifications compared to previous generations that had, for example, increased access to pensions. The growth in individual responsibility dovetailing with the growth in financial products and services, including those in the Alternative Financial Services sector, has resulted in a higher risk than ever for making financial decisions. As a result of the recent global financial crisis and subsequent growth in income and wealth inequality to unprecedented levels in recent U.S. history, there is growing recognition that people need stronger financial capability to avoid and recover from financial difficulties and poverty (Miller, Reichelstein, Salas, & Zia, 2014; Mitchell & Lusardi, 2015).

### 1.2 The intervention

There is a growing academic and public policy interest in helping people gain financial capability. Researchers are testing financial capability interventions with adults, children, immigrant populations, and other groups (Theodos et al., 2015; Batty, Collins, & Odders‐White, 2015; Huang, Nam, & Sherraden, 2013; Curley & Robertson, 2014). These interventions use various methods to increase financial education combined with financial access. The interventions differ in their methods of both financial education and financial access and their method for combining them, but they share this coordinated combination. For example, interventions to help parents learn to save money include financial education and access to college savings account for their child (Huang et al., 2013). Policy makers are showing increased interest in these interventions and implementing new policies designed to increase financial capability. For example, the U.S. states of Maine and Nevada have started statewide financial capability and asset building programs (Clancy, Sherraden, & Beverly, 2015). Countries are creating national strategies on financial capabilities, such as the countries within the United Kingdom, which set out a clear description of the problem, and define clear goals for specific populations and geographic areas (Bagwell, Hestbaek, Harries & Kail, 2014; Kempson, 2009). As outcomes, studies measure financial behaviors, such as setting aside savings as emergency or short‐term savings (Azurdia & Freeman, 2016, Birkenmaier, Curley, & Kelly, 2014; Collins & Urban, 2016; Huang et al., 2013; Skimmyhorn, 2012; Theodos et al., 2015), financial management (e.g., keeping records of expenses and income, paying bills on time, and using a budget; Theodos et al., 2015), improving credit (Birkenmaier, et al., 2014; Theodos et al., 2015), participation in retirement savings plans (Duflo & Saez, 2003; Duflo, Gale, Liebman, Orszag, & Saez, 2007) and saving for an asset, such as children's college education (Han, Grinstein‐Weiss, & Sherraden, 2009; Huang et al., 2013, Sherraden, Johnson, Guo & Elliott, 2011). Some studies are also interested in financial knowledge (Azurdia & Freeman, 2016; Han et al., 2009; Theodos et al., 2015) and financial mindset (i.e., attitudes, motivation, and decision‐making; Skimmyhorn, 2012; Theodos et al., 2015). These outcomes all have the potential to impact a person's financial behaviors, which in turn impacts financial well‐being, or the ability of a household to “fully meet current and ongoing financial obligations, feel secure in their financial future, and make choices that allow them to enjoy life” (Consumer Financial Protection Bureau, 2015).

### 1.3 How the intervention might work

There is a growing awareness that financial knowledge, while necessary for optimal financial choices and behaviors, is insufficient by itself in today's world (Austin & Arnott‐Hill, 2014; Fernandes, Lynch, & Netemeyer, 2014; Hastings, Madrian & Skimmyhorn, 2013; Miller et al., 2014; Mitchell & Lusardi, 2015). Results of two meta‐analysis studies focused on financial education efforts alone suggest that by itself, financial education has weak effects on financial behavior (Fernandes et al. 2014; Miller et al., 2014). A third recent meta‐analysis found that the impacts of financial education are highly heterogeneous (Kaiser & Menkhoff, 2016).

**Figure 1:**
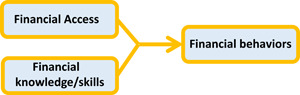
Framework of financial capability

Instead, a focus on the combination of *financial knowledge and skills, and access to appropriate financial products and services* (Sherraden, 2013), or financial capability, has demonstrated promise to result in financial behaviors and financial access that facilitate financial well‐being (Theodos et al., 2015; Collins & Urban, 2016; Huang et al., 2013; Curley & Robertson, 2014). This combination is grounded in Sen (1999) and Nussbaum's (2007) theoretical work on capability, which postulates that people's choices reflect both their knowledge and their real opportunities within their lived environment. Capability incorporates people's internal capabilities (abilities, knowledge, and skills) with external capabilities (e.g., the range of opportunities available through products, services, and institutions). Internal and external capabilities interact to further develop one's internal capabilities (Nussbaum, 2011, p.21). Applying these concepts to financial capability means focusing on the financial decisions people make based on their innate ability, knowledge, skills, as well as the opportunities afforded them through their environment. Their innate ability to demonstrate financial behaviors is a result of the interaction of their internal and external capabilities, and growth of their internal capability through such interaction (Sherraden, 2013). To improve one's financial behaviors, a focus on both internal capabilities through financial education and external capabilities through the financial products and services available to them is needed. As shown in Figure 1 below, the financial capability framework recognizes that both financial education and access are determinants of an individual's financial behaviors, and that the interaction of the two allows individuals to apply their knowledge and skills through their financial behaviors, such as the degree to which they save money for emergencies or retirement, pay bills on time, and invest in assets that appreciate in value (Huang, Nam, Sherraden, & Clancy, 2014). The interventions that use both elements also utilize important elements identified by the World Bank as essential to better financial interventions. By combining knowledge and financial access, interventions are “targeted and relevant”, provided at a “teachable moment” when the information is useful because it will be applied in a real‐world setting with a financial product or service that is useful currently or in the near future for the participant, and “gives exposure to information over the longer‐term” through the use of a product or service (Lundberg & Mulaj, 2014).

Interventions designed to increase financial capability are diverse, yet designed to both strengthen financial knowledge and strengthen access to appropriate financial products to impact behavior. Financial knowledge interventions involve a diverse range of types, goals, and delivery models. Some financial knowledge interventions utilize a set curriculum (Birkenmaier et al. 2014), while others are tailored to the client's situation (Huang et al. 2013), expressed goals and interest areas (Theodos et al., 2015), or are specific to a financial product or service (Azurdia & Freeman, 2016; Collins & Urban, 2016). Financial knowledge interventions may have the goal of increasing general financial knowledge (Han et al., 2009), financial knowledge specifically related to a particular client situation (Theodos et al, 2015), or financial knowledge about a particular type of product or service (Azurdia & Freeman, 2016; Collins & Urban, 2016). Delivery models include one‐on‐one financial education (Azurdia & Freeman, 2016; Theodos et al., 2015), online financial education (Collins & Urban, 2016), and classroom‐based financial education (Birkenmaier et al. 2014). Within interventions designed to improve financial capability, these financial knowledge elements are paired with a financial access element, such as debt or credit interventions for a particular client situation (Theodos et al., 2015), a child education savings account (Huang et al. 2013), savings and checking accounts at banks and credit unions (Birkenmaier et al. 2014), retirement saving plans (Collins & Urban, 2016), matched savings accounts for asset development (Han et al., 2009), or emergency savings accounts (Azurdia & Freeman, 2016), among others. The range of financial capability behaviors impacted includes savings, investment, record keeping, and loan repayment behaviors (Miller et al., 2014).

An important difference among interventions, however, is whether the intervention includes only financial education, or whether financial education is linked to financial products and services onto which participants can act to better their financial situation with their newfound knowledge. This difference is important. Interventions that include only financial education and are not designed to increase participant access to appropriate financial products and services that allow them to act on their knowledge assume that knowledge alone can result in financial behavior change. Indeed, a prior review found that financial education alone had weak effects on behavior (Fernandes et al. 2014).

Financial capability interventions utilized for this review combine financial education and financial access and thus capitalize on the interaction of the two elements to affect financial behavior.

### 1.4 Why it is important to do the review

Three prior reviews have been conducted on interventions intended to improve at least one aspect of financial capability.

Fernandes et al. (2014) examined the effects of financial literacy and financial education interventions on financial behaviors. Although the review methods were not clearly reported, and the inclusion criteria were not well defined, the authors appear to have included 168 studies (published and unpublished), with 90 of those being studies that manipulated financial literacy with some education intervention and the remaining being studies that measured financial literacy (correlational studies). Fifteen of the 90 intervention studies used a randomized design, and the others used a quasi‐experimental or pre‐post design. They found that financial education interventions had weak effects on financial behavior, especially in low‐income samples. They found that financial literacy explains only 0.1% of the variance in financial behaviors studied, with weaker effects in low‐income samples.

Kaiser and Menkhoff (2016) also conducted a review of financial literacy and financial education interventions on financial behaviors. The authors included 126 impact evaluation studies (published and unpublished) that were designed to impact financial knowledge or behaviors, and that report on financial literacy and/or financial behavior outcomes. Forty‐four percent of included studies overlap with the Fernandes et al (2014) study, 83% of the studies were of classroom financial education, 8% were of online financial education, 2% were individualized counseling interventions, and 7% were informational and behavioral nudges. Results suggest that financial education intervention impacts are less effective for low‐income clients and for those in low‐ and lower‐middle income countries. They also found that offering financial education at “teachable moments” and increasing educational intensity increases the success of financial education efforts.

Miller et al. (2014) took a seemingly broader approach to their review, including any intervention that would impact financial knowledge, attitudes, and/or behaviors. They identified 188 studies via their search of one electronic database (EconLit), prior literature reviews, studies completed within the World Bank, and websites likely to include relevant studies. However, the authors reported that “in order to reduce the number of studies to a manageable size … only articles from peer‐reviewed journals were included from Econlit, for the period January 2009 to September 2013” (p. 7). Despite their seemingly broader inclusion criteria related to the interventions of interest, their meta‐analyses reported on outcomes of financial education interventions from a small number of studies on the following outcomes: savings behavior (*n* = 6), retirement savings (*n* = 5), loan defaults (*n* = 4), and record keeping (*n* = 5). Findings indicate that financial education interventions had a positive and statistically significant mean effect on retirement savings (effect sizes [ES] = 0.08; 95% confidence interval [CI], 0.01, 0.16), but a null or negative and nonstatistically significant mean effect on savings (ES = 0.03; 95% CI, 0.00, 0.06), record keeping (ES = 0.04; 95% CI, 0.00, 0.09), and loan default (ES = –0.02; 95% CI, –0.06, 0.02). The authors did not provide any analysis or discussion regarding whether the interventions had any clinical or practical significance for any of the outcomes. It is also important to note that the authors did not provide any details about how they calculated effect sizes or even which effect size statistic they were reporting. Overall, the reporting of the eligibility criteria and methods used to search, select, and extract data from studies was not clear. Miller et al. (2014) may have included interventions that encompassed financial literacy and education and access, but the authors' inclusion criteria were not clear, and they did not describe the types of interventions they included, aside from referring to them as “financial education.”

While these reviews provide some evidence related to the effects of financial capability interventions, serious evidence gaps remain.

Fernandes et al. (2014) and Kaiser and Menkhoff (2016) included only financial education or literacy efforts without financial access (i.e., no link to a financial product or service). Therefore, the interventions were not designed to increase participant access to appropriate financial products or service that allows them to act on their knowledge. Similarly, Miller et al. (2014) appear to have focused on financial literacy interventions; if they did include financial capability interventions, they made no distinction between these the different types of interventions. In addition to this limitation, the Miller et al. (2014) study also has some shortcomings that limit its usefulness in policy formation. The review had an insufficient search strategy by using a limited number of databases, and therefore, the review may have missed potentially relevant studies. The review does not sufficiently describe the types of evidence included in the review and does not assess the risk of bias in the included studies. Thus it remains unclear whether interventions that combine financial literacy with financial access are effective.

As interventions to improve financial capability move forward, more evidence is needed about the effects of interventions that combine financial education and financial access. It is important that practitioners, policy makers, and stakeholders have access to synthesized evidence of effects of approaches to improve financial capability that combine both elements to make informed decisions, rather than relying on results of individual studies. A systematic review could inform practice decisions by providing evidence about the components of a financial capability intervention that is essential to effect participant financial outcomes. Policy decisions about essential program design elements required for funding could also be guided by evidence.

## 2 OBJECTIVES

The purpose of this review is to examine and synthesize evidence of effects of interventions designed to improve financial capability that combines financial education and access to financial products and/or services. Results of this review should inform related practice and policy. The following is the research question for this project: What are the effects of interventions designed to improve financial capability on financial behavior and financial access?

## 3 METHODOLOGY

### 3.1 Characteristics of the studies relevant to the objectives of the review

While definitions, terms, and practices vary across studies, for purposes of this review, financial capability is defined as “*a consumer's ability to apply financial knowledge and perform desirable financial behaviors to achieve financial well‐being*” (Xiao & O'Neill, 2014, November). Studies eligible for this review will examine the effectiveness of interventions designed to improve financial capability that uses a combination of financial education or information and access to a financial product or service.

Two U.S. studies provide examples of research on financial capability interventions that combine financial education and access. A study by Huang et al. (2013) analyzed data from a statewide randomized social experiment to test a Child Development Account (CDA) program that encourages families to accumulate assets for their children's future using the existing 529 College Savings Plan in Oklahoma. CDAs are savings accounts for children that provide a structured opportunity to save and accumulate assets by providing incentives, financial information and access to tax‐advantaged savings programs operated by U.S. state governments to encourage savings for future college costs. Participants (children) received an individual deposit in a state‐owned 529 account, opened automatically for each child at birth unless participant (via their parents) opted out of this option. Parents were also provided the opportunity to open their own 529 college savings account for their child. In the process, parents were provided financial information about saving and saving for college. Results indicate that the interactive effects between financial knowledge and opening a participant‐owned 529 account are statistically significant (*b* = 0.85, standard error = 0.40), as compared to a control group. The finding suggests that the effect of financial knowledge on financial decisions related to college savings is moderated by financial access.

Collins & Urban (2016) conducted a randomized field study on the role of information and access to retirement savings plans on retirement savings. Their study was a randomized field study in which participants, which were employees of credit unions, were offered 10 hr of online financial education about retirement savings, in addition to access to their employers' retirement savings plans. They measured a variety of participant financial behaviors, such as using a budget or spending plan, using employer‐based financial benefits (such as retirement savings and other benefits), nonemployer‐based retirement accounts (such as individual retirement accounts [IRAs]), and saving for emergencies. They found that the combination of financial education and access changed financial behaviors; use of nonemployer‐based retirement savings (IRAs), employer benefits, emergency savings, and employer‐sponsored retirement savings all increased, as compared to a comparison group.

### 3.2 Criteria for including and excluding studies

#### 3.2.1 Types of study designs

To mitigate threats to internal validity, studies must use a prospective randomized controlled trial (RCT) or quasi‐experimental (QED) research design with parallel cohorts. Studies using single‐group pre‐post test design (SGPP), or single subject design (SSD), or historical comparisons will be excluded.

#### 3.2.2 Types of participants

Included: Financial and economic policies and practices can be quite different for high‐income compared to low‐ and middle‐income countries. Therefore, the focus of this review will be on financial capability interventions in high‐income countries. Studies must have been conducted with participants in any of the 35 member countries of the Organization for Economic Co‐Operation and Development (OECD). Studies with participants of all ages will be included.

Excluded: Studies conducted in non‐OECD countries. Studies will also be excluded that teach financial education only or facilitate financial access only.

#### 3.2.3 Types of interventions

To be eligible for this review, the intervention must include a financial education component and a financial product or service.

To meet the criteria for delivering financial education, interventions must deliver information about: (a) a variety of general financial concepts and behaviors (such as using a formal education curriculum that covers the time value of money and the importance of keeping financial records); (b) a specific financial topic (such as a formal or informal one‐time education session about savings or homeownership); (c) a specific product (such as retirement savings accounts or savings accounts that can be used for emergency savings); and/or (d) a specific service, such as the value of prepurchase homeownership counseling to gain access to low‐cost financing. Information about a specific product or a specific company will also meet the criteria, such as in the case when employer‐based financial education is focused on educating their employees about their retirement plan, and options for investing within their plan.

To meet the criteria for access to a financial product or service, interventions must facilitate access to: (a) a child development account (used for post‐secondary education or training, or another type of asset purchased at age 18 or older); (b) a retirement account through an employer; (c) a “second chance” checking account (for persons listed in a consumer reporting bureau after having insufficient funds for a check; (d) a matched savings account (to pay debts to build assets); (e) a financial service, such as debt or credit counseling; (f) a bank account; or (g) a specialized savings vehicle, such as Certificate of Deposit (CD). Facilitating access includes linking participants to products or services that are tailored toward the population of participants (such as financially vulnerable populations, or employees eligible for employer‐provided retirement benefits). The interventions could facilitate the access by signing participants up for the product or service (such as a savings account), delivery service as part of the intervention (debt or credit counseling), and/or provide ongoing interpersonal support to make it possible for participants to maintain access to a product or service.

Studies that use multi‐component interventions will be included as long as two of the components are financial education or information and access to a financial product or service. Studies that describe interventions that teach financial education only or financial access only will be excluded from this review.

#### 3.2.4 Types of outcome measures

Studies must measure at least one of the following primary outcomes. These outcomes reflect financial behavior:

P1. *Behavior change.* Behavior change refers to changes in financial behaviors of participants, such as opening a savings or checking account, owning a retirement or College Savings account, active use of savings or checking accounts, increased frequency of savings, change from using predatory financial products to mainstream financial products, purchase of an asset, reviewing credit report, etc.

P2. *Financial outcomes*. Financial outcomes refer to implications of behavior change, such as higher fund balance in a savings or checking account, higher net worth, lower debt, and improved credit scores.

If studies measure one of the above primary outcomes, data will be extracted on the following secondary outcome:

S1. *Adverse effects.* When reported, all adverse effects reported by the primary study authors will be included. These will be used descriptively. An example of an adverse effect that could be reported is that families experience a decrease in their material well‐being (e.g., experience insecure housing or food) because they are saving money for their child's college education rather than spending it on necessities. However, based on our preliminary review of studies, we do not expect studies to report adverse effects.

Measurement of above outcomes may be conducted using standardized or unstandardized instruments and may be self‐ or other‐reported or researcher administered measures—the reviewers will not exclude measures based on the type of measure but may pool effects based on the type of measure used (e.g., observational measures will be pooled with observational measures). To be included in the meta‐analysis, primary study authors must report enough information to calculate an effect size. If sufficient information to calculate an effect size is not provided, every effort will be made to contact primary study authors and request the necessary information.

#### 3.2.5 Types of comparison conditions

For RCT and QED studies, waitlist control, no treatment, treatment‐as‐usual, and alternative treatment groups will be considered acceptable comparison groups. The reviewers will code and report each study's comparator condition and, if there are a sufficient number of studies and significant heterogeneity is found, the type of comparator condition will be examined as a moderator.

#### 3.2.6 Duration of follow‐up

The reviewers will include measurement points at post‐test and all follow‐up time points. We will synthesize studies that report similar follow‐up time points (i.e., up to 3 months, 3−6 months, 6−12 months, >12 months) if there are more than two studies that report sufficient data

#### 3.2.7 Types of settings

All settings are eligible for inclusion.

#### 3.2.8 Other

*Language.* The reviewers will make attempts to have non‐English articles translated; however our resources for doing so are very limited. If studies are not available in English or we cannot translate the studies to English, they will be excluded and noted accordingly in the excluded studies table.

### 3.3 Search strategy

We will attempt to identify and retrieve both published and unpublished studies through a comprehensive search that includes multiple electronic databases, grey literature sources, and reference lists of reviews and relevant studies (Kugley et al., 2017). The search strategy and results of the search will be documented in sufficient detail to produce a flow chart.

1. Electronic databases: ABI/INFORM; Academic Search Complete; Bloomberg Professional Service; Business Source Premier; Database of Research on International Education; Dissertation & Theses Global; EconLIt; Education; ERIC; Education source; JSTOR; PAIS International; PsychINFO; Public Affairs Index; Social Science Research Index; Social Sciences Citation index; Social Work abstracts; Sociological Abstracts.
2. Trial registries: We will search the following trial registries: Clinicatrials.gov and the International Clinical Trials Registry Platform (WHO; https://www.who.int/ictrp/network/en/).
3. Website and online sources: Our search for unpublished studies will include relevant websites, to include the website “finlitedu.org” for studies completed by the World Bank, and the OECD, Global Partnership for Financial Inclusion, and Alliance for Financial Inclusion websites.
4. The reference lists from prior reviews and included studies will be reviewed for potential studies. We will also conduct forward citation searching using Google Scholar to search for studies citing included studies.
5. Authors of prior studies will be contacted in an attempt to obtain unpublished studies, studies in process and published studies missed in the database search.

### 3.4 Search terms and keywords

We will use combinations of terms related to the intervention and study design to search the electronic databases. Database‐specific strategies will be explored for each database in consultation with a librarian at Saint Louis University, including the use of truncation and database‐specific limiters and thesauri will be consulted to employ more precise search strategies within each database. Below are examples of the types of terms we anticipate using:

Intervention: (financial OR economic OR bank) AND (education OR knowledge OR literacy), AND (capability OR access OR inclusion OR exclusion OR attachment)

AND

Report type: (evaluation OR intervention OR treatment OR outcome OR program OR trial OR experiment OR “control group” OR “controlled trial” OR quasi‐experiment” OR random*)

### 3.5 Data extraction and study coding categories

For all studies that pass the eligibility screening process described above, two reviewers will independently code all eligible studies using a structured data extraction form (see Appendix B). Multiple reports on individual studies will be collated. The data extraction form includes items related to bibliographic information and source descriptors; methods and procedures; context, nature, and implementation of the intervention; sample characteristics; and outcome data needed to calculate effect sizes.

wo trained coders will code all included studies. If greater than 20 studies are eligible for inclusion in the review, a third coder may be recruited to assist with coding. Coders will pilot test the code form together using diverse types of studies and will discuss any items that are unclear and ensure mutual understanding of all items. Following pilot testing of the form, two coders will independently code 100% of the included studies. Coders will compare coding and will identify and discuss discrepancies, which will be resolved through consensus. If consensus cannot be reached between the two coders, a third member of the review team will be consulted to resolve the discrepancy. Initial discrepancies will be recorded and inter‐rater reliability will be reported. In addition to comparing codes between coders, we will also compare the magnitude and direction of effects as presented in the review with how data is presented in the primary study to check for agreement.

### 3.6 Description of methods used in primary research

To mitigate threats to internal validity, studies must use a prospective RCT or QED research design with parallel cohorts. Studies using SGPP, or SSD, or historical comparisons will be excluded. We do not plan to include qualitative research. Studies will use a wide variety of statistical methods for the analysis.

### 3.7 Criteria for determination of independent findings

We are interested in two primary outcome constructs: behavior change and change in access to a financial product or service. We anticipate that some included studies may use multiple measures for each outcome, multiple reports of the same outcome measure, multiple follow‐up time points, more than one intervention, and possibly more than one counterfactual condition. These circumstances create statistical dependencies that violate assumptions of standard meta‐analytic methods. In order to ensure independence of study‐level effect sizes, we will include only one effect size estimate from each independent sample in each meta‐analysis.

For cases in which a study uses multiple measures (i.e., observation and a standardized instrument) of the same construct, we will code data for each measure and create a study‐level average across the measures. In cases of multiple reports on the same outcome (i.e., parent and child report), we will code data for each report and conduct separate meta‐analyses (i.e., similar types of reporters will be pooled). In cases where multiple points of follow‐up are provided, we will code follow‐up points to conduct a separate analysis for effect sizes comparing studies with similar points of follow‐up. In the case of multiple counterfactual conditions, we will select the comparison condition that is most similar to those in the other included studies. For studies in which multiple interventions are tested, we will select the intervention that meets the inclusion criteria for this review. If there is more than one intervention in a study that meets the criteria for this review, we will combine the intervention groups.

### 3.8 Details of study coding categories

For all studies that pass the eligibility screening process described above, two reviewers will independently code all eligible studies using a structured data extraction form (see Appendix B). Multiple reports on individual studies will be collated. The data extraction form includes items related to bibliographic information and source descriptors; methods and procedures; context, nature, and implementation of the intervention; sample characteristics; and outcome data needed to calculate effect sizes.

Two trained coders will code all included studies. If greater than 20 studies are eligible for inclusion in the review, a third coder may be recruited to assist with coding. Coders will pilot test the code form together using diverse types of studies and will discuss any items that are unclear and ensure mutual understanding of all items. Following pilot testing of the form, two coders will independently code 100% of the included studies. Coders will compare coding and will identify and discuss discrepancies, which will be resolved through consensus. If consensus cannot be reached between the two coders, a third member of the review team will be consulted to resolve the discrepancy. Initial discrepancies will be recorded and inter‐rater reliability will be reported. In addition to comparing codes between coders, we will also compare the magnitude and direction of effects as presented in the review with how data is presented in the primary study to check for agreement.

### 3.9 Risk of bias

Two review authors will independently assess the risk of bias in all included studies using the Cochrane Collaboration's risk of bias tool (Higgins et al., 2011). We will assess risk of bias for each of the six following domains: allocation, blinding, complete outcome data, selective reporting, and other potential sources of bias (i.e., researcher allegiance, funding source). Each study will be coded as “low”, “high”, or “unclear” risk of bias on each of the domains. Following independent coding by two authors, coders will meet to identify any discrepancies, and all discrepancies will be resolved through consensus. If consensus cannot be reached between the two reviewers, a third member of the review team will be consulted.

Risk of bias in each domain will be reported within and across studies in the results section using narrative and graphs. We anticipate that most studies included in this review will be at high risk of bias in terms of allocation and blinding; thus, we do not plan to restrict analyses based on the risk of bias in any domain. We plan to present all included studies, provide the evidence and source used for the judgment, and provide a narrative discussion of the risk of bias to include discussion of the potential limitations of the review as well as implications of bias in the interpretation of the results in the Discussion section of the review.

### 3.10 Statistical procedures and conventions

We will conduct descriptive analyses on variables of interest from all included studies to provide information regarding

- study participants (e.g., subgroups, gender, race/ethnicity, income level, age);
- settings where studies are situated (e.g., community nonprofits, schools, geographical location/country);
- relevant intervention characteristics (e.g., type/duration of financial education, type of financial product(s)); and
- risk of bias across RCT and QED studies on each domain.

Following descriptive analysis, we will estimate the effect sizes for each outcome in each included study. For RCT and QED studies, we will calculate the magnitude of effect using the standardized mean difference effect size with Hedges' *g* correction for continuous outcomes and odds ratios for outcomes presented as dichotomous variables. We anticipate that outcomes within a category will be measured using similar metrics and therefore, effect sizes within each category will only use either Hedges' *g* or odds ratio metrics in the analysis. If, however, outcomes within one of the outcome categories are measured in different metrics, we will convert them to Hedges' *g* using CMA software. When studies use non‐standard designs (e.g., clustering), we will follow procedures per the What Works Clearinghouse Procedures and Standards Handbook version 3.0 (Institute of Education Sciences, 2014). All effect sizes will be coded so that a positive effect size is indicative of the treatment group positively outperforming the comparison group on the outcome. If primary studies do not report sufficient data to calculate an effect size, we will contact the study authors and request the data. If we are unable to obtain the data, we will include the study in the review, but it will not be included in the meta‐analysis. We interpret a statistically nonsignificant *p* value (e.g. larger than 0.05) as a finding of uncertainty unless confidence intervals are sufficiently narrow to rule out an important magnitude of the effect.

Following the estimation of individual study‐level effects, we will conduct separate meta‐analyses to pool studies for each outcome construct. A weighted mean effect will be calculated by weighting each study‐level effect size by the inverse of its variance. Random effects statistical models will be used throughout unless a compelling case arises for fixed effect analysis. RCT studies will be pooled separately from QED studies; however, if RCT and QED studies are found to be homogenous, studies will be pooled to allow for greater statistical power.

Following the estimation of summary effects, we will conduct a test of homogeneity (*Q* test) to compare the observed variance to what would be expected from sampling error. The I2 statistic will also be used to describe the percentage of total variation across studies due to the heterogeneity rather than chance. We will also construct a forest plot displaying study‐level mean effect sizes and 95% confidence intervals for the included studies to provide opportunity for visual analysis of the precision of the estimated effect sizes, detection of studies with extreme effects, and information regarding the heterogeneity of studies. Publication bias will be assessed using funnel plots and the Egger's test, both will be conducted in CMA version 3.0.

Provided there are a sufficient number of studies, we will conduct moderator analysis to examine whether characteristics of the study methods and interventions may be associated with effect size. The approach to moderator analysis will be dependent upon the available data. If there are sufficient studies available, meta‐regression will be used for continuous outcomes and the analog to the Analysis of Variance will be used for categorical variables. Moderating variables of interest include study design (RCT and QED), publication status (published and unpublished), dosage and duration of intervention (continuous variable), geographic location (e.g., United States, Canada, Europe, Norwegian Countries, Asia), socio‐economic status (SES) of sample (low SES and range of SES), age (children, college age, adults, older adults/retired persons), and type of intervention (child development account, individual development account, retirement savings, youth‐employment savings, second chance savings accounts, retirement accounts, prepurchase home buying education, and credit counseling). Sensitivity analysis will be conducted to examine the potential effects of outliers. Analyses will be conducted using Comprehensive Meta‐Analysis 3.0 (CMA). (Table 1)

**Table 1:** Financial capability studies

Study	0utcome	Results
Huang et al. 2013	Financial asset	1. Being assigned to the treatment group increases the odds of holding a child development account 33.5 times.
Azurdia & Freeman, 2016	Savings	1. SaveUSA participants had an average of $522, or 30 percent, more saved than regular tax filers, and were eight percentage points more likely to hold any kind of savings 42 months after program enrollment

### 3.11 Treatment of qualitative research

We do not plan to include qualitative research.

## 4 ROLES AND RESPONSIBILITIES

- Content: Julie Birkenmaier will be responsible for the substantive content related to financial capability. Birkenmaier is a noted expert on financial capability as the author and coauthor of numerous publications on the topic, to include coauthoring of *Financial Capability and Asset Building in Vulnerable Households* (under development, Oxford University Press), and *Financial Capability and Asset Development: Research, Education, Policy and Practice* (Oxford University Press). Youngmi Kim will assist with the substantive content as well. She is a noted expert on interventions that include financial capability, including asset‐building interventions known as Individual Development Accounts (IDAs) and Child Development Accounts (CDAs).
- Systematic review methods: Brandy Maynard is a noted expert in systematic review methods. Maynard has completed and published multiple systematic reviews/research syntheses. In addition, Maynard has been trained in C2 methods and is actively involved in C2— she has produced two Campbell reviews and is a coauthor on two additional reviews, is an editorial board member of the ECG, is a C2 methods trainer, and has been elected as co‐chair of the social welfare group.
- Statistical analysis: Brandy Maynard will be primarily responsible for statistical analysis. and Birkenmaier and Kim will assist. While Birkenmaier and Maynard have been trained in meta‐analytic techniques, Maynard has conducted several meta‐analyses.
- Information retrieval: Maynard will take primary responsibility for information retrieval, and be assisted by Birkenmaier and Kim. Review authors may also enlist trained graduate students or colleagues to assist in the search and data extraction process. They will also consult with information retrieval specialists within Saint Louis University in the planning and execution of the search strategy.

## 5 SOURCES OF SUPPORT

We have not received any financial support to conduct this review. Our application to C2 for funding was rejected.

## 6 DECLARATIONS OF INTEREST

The review team declares no potential conflicts of interest.

## 7 PRELIMINARY TIMEFRAME

The review will be completed within 1 year of the approval of the protocol.

## 8 PLANS FOR UPDATING THE REVIEW

If enough new studies have been published in 3 years, we will consider updating the review.

## 9 AUTHOR DECLARATION

### 9.1 Authors' responsibilities

By completing this form, you accept responsibility for preparing, maintaining and updating the review in accordance with the Campbell Collaboration policy. Campbell will provide as much support as possible to assist with the preparation of the review.

A draft review must be submitted to the relevant Coordinating Group within 2 years of protocol publication. If drafts are not submitted before the agreed deadlines, or if we are unable to contact you for an extended period, the relevant Coordinating Group has the right to de‐register the title or transfer the title to alternative authors. The Coordinating Group also has the right to de‐register or transfer the title if it does not meet the standards of the Coordinating Group and/or Campbell.

You accept responsibility for maintaining the review in light of new evidence, comments and criticisms, and other developments, and updating the review at least once every 5 years, or, if requested, transferring responsibility for maintaining the review to others as agreed with the Coordinating Group.

### 9.2 Publication in the Campbell Library

The support of the Coordinating Group in preparing your review is conditional upon your agreement to publish the protocol, finished review, and subsequent updates in the Campbell Library. Campbell places no restrictions on publication of the findings of a Campbell systematic review in a more abbreviated form as a journal article either before or after the publication of the monograph version in Campbell Systematic Reviews. Some journals, however, have restrictions that preclude publication of findings that have been, or will be, reported elsewhere and authors considering publication in such a journal should be aware of possible conflict with the publication of the monograph version in Campbell Systematic Reviews. Publication in a journal after publication or in press status in Campbell Systematic Reviews should acknowledge the Campbell version and include a citation to it. Note that systematic reviews published in Campbell Systematic Reviews and co‐registered with Cochrane may have additional requirements or restrictions for co‐publication. Review authors accept responsibility for meeting any co‐publication requirements.

## APPENDIX A

**Interventions Designed to Improve Financial Capability by Improving Financial Behavior and Financial Access: A Systematic Review**

## APPENDIX B Coding form

***Outcomes***

(By intervention type) – see below

**Continuous and dichotomous outcomes**

**Table:**

Timing of measurement (during tx, end of tx, 3 month, etc.) CAN ADD if needed	Tx Baseline Mean	Tx Baseline SD	Tx baseline N	Tx Post Mean	Tx Post SD	Tx Post N	Control group Baseline Mean	Control group SD	Control group N	Values for t, F, other
										
										
										
										

Continuous Construct ID: 1 = Savings Amount; 2 = Savings rate; 3 = Fund balance; 4 = Credit score; 5 = debt; 6 = value of asset; 7 = other.

Dichotomous Construct ID: 1 = Open account; 2 = Active use of accounts; 3 = Use new product; 4 = budget; 5 = lower debt; 6 = purchase of asset; 7 = other.
